# Impact of cervical and global spine sagittal alignment on cervical curvature changes after posterior cervical laminoplasty

**DOI:** 10.1186/s13018-022-03421-w

**Published:** 2022-12-02

**Authors:** Xiang-Yu Li, Yu Wang, Wei-Guo Zhu, Chao Kong, Shi-Bao Lu

**Affiliations:** 1 Department of Orthopedics, Xuanwu Hospital, Capital Medical University, No.45 Changchun Street, Xicheng District, Beijing, China; 2 National Clinical Research Center for Geriatric Diseases, Beijing, China

**Keywords:** Spino-pelvic sagittal balance, Cervical sagittal alignment, Laminoplasty

## Abstract

**Objective:**

To analyze the correlation between the changes in cervical curvature and the sagittal parameters of spino-pelvic and clinical efficacy after posterior laminoplasty (LP).

**Methods:**

The patients with cervical spondylosis treated with LP from June 2018 to December 2020 were reviewed. The preoperative and follow-up spine full-length films were measured. The measured data included C2–C7 Cobb angle, C2–7 sagittal vertical axis (SVA), T1 slope (T1S), pelvic incidence, sacral slope (SS), pelvic tilt (PT), lumbar lordosis (LL), thoracic kyphosis (TK), and C7-SVA. Japanese Orthopaedic Association (JOA) score and neck disability index (NDI) score were recorded before surgery and follow-up.

**Results:**

There were 56 patients in this study. There were no significant differences in spino-pelvic sagittal parameters before and after surgery; however, the JOA score significantly improved. The changes in postoperative cervical lordosis correlated with SS, PT, LL, T1S, and C7-SVA (*P* < 0.05). Regression analysis showed that T1S and C7-SVA were associated with reducing cervical lordosis (*P* = 0.021 and *P* = 0.001, respectively). Patients with larger T1S combined with larger C7-SVA had more cervical lordosis loss, poor JOA improvement, and high postoperative NDI scores (*P* < 0.001, *P* = 0.018, and *P* < 0.001, respectively).

**Conclusion:**

Patients should be examined with full-length spine film before surgery to evaluate the cervical and spino-pelvic sagittal balance. T1S and C7-SVA correlated with changes in cervical sagittal alignment after LP.

**Level of evidence:**

III.

## Introduction

Cervical spondylotic myelopathy (CSM) is a degenerative disease that leads to spinal cord dysfunction; it can be caused by cervical bone hyperplasia, cervical disk herniation, ligament hypertrophy, or cervical canal stenosis. Common symptoms include neck and shoulder pain, numbness of upper limbs, muscle atrophy, difficulty walking, limb paralysis, defecation dysfunction, and even paralysis [1].

Surgery should be considered for patients with multilevel CSM who fail conservative treatment. Currently, laminoplasty (LP) is the primary surgical method. This procedure is relatively simple, has a wide range of applications, and achieves adequate decompression and symptomatic relief. However, LP outcomes are related to maintaining the postoperative cervical lordosis angle. Better cervical lordosis can ensure postoperative spinal cord drift and adequate indirect decompression. For patients with postoperative cervical lordosis loss, the effect of decompression may be compromised, leading to less favorable outcomes [2, 3].

LP is a decompression procedure without fusion that maintains the range of motion of the cervical spine and preserves the integrity of adjacent tissue structures [4, 5]. However, the preoperative cervical sagittal imbalance is related to the sagittal cervical imbalance after LP [6, 7]. Studies found that preoperative cervical sagittal parameters can predict the loss of cervical lordosis after LP. Suk et al. found that preoperative cervical lordosis < 10° is related to the occurrence of postoperative kyphosis [8]. Kim et al. demonstrated that patients with a more significant T1 slope (T1S) had more lordosis loss after LP [9]. There has been increasing attention to the relationship between sagittal spinal parameters and changes in cervical curvature after LP; T1S > 20° and C2–7 sagittal vertical axis (SVA) > 22 mm correlated with loss of cervical lordosis after LP [10]. However, these studies did not include full-length spine films, and the relationship between the change of cervical sagittal parameters and spino-pelvic sagittal parameters after LP requires further study.

Research on cervical curvature changes after LP focused on the influence of local sagittal parameters of the cervical spine. There is a lack of correlation between research focused on overall sagittal spine parameters and changes in cervical curvature after LP. Therefore, this study was designed to determine the relationship between overall sagittal spino-pelvic parameters and changes in cervical curvature after LP and to determine the correlation between cervical curvature and outcomes.

## Methods

### Patient information

Hospitalization data of CSM patients from June 2018 to December 2020 were retrospectively analyzed. We recorded gender, age, follow-up time, and surgical segment. Inclusion criteria were as follows: (1) cervical spondylosis with multilevel cervical lesions (three or more segments) treated with LP; (2) follow-up time > 6 months; (3) full-length spine X-rays before surgery; and (4) complete follow-up. Exclusion criteria were as follows: (1) spinal tumor trauma; (2) history of nervous system diseases; (3) spinal cord injury; (4) history of previous cervical surgery; and (5) requirement for surgical revision.

### Surgical method

After induction of general anesthesia, the patient was positioned prone on the operating table and fixed with a Mayfield head holder to place the neck in a relatively extended position. An incision was made in the middle of the posterior square of the neck. We exposed the posterior structure of the cervical spine layer by layer. The paraspinal muscles of patients were separated to expose the lamina. One side of the lamina is 2 mm away from the inner side of the articular process; the entire lamina was removed, and on the other side, bites were taken off of the lamina as a hinge. We supported the lamina with a titanium plate and fixed it with screws to maintain the open space of the spinal canal. Finally, we placed paravertebral drainage and closed the fascia and skin layer by layer.

### Spino-pelvic parameters

**Fig. 1:**
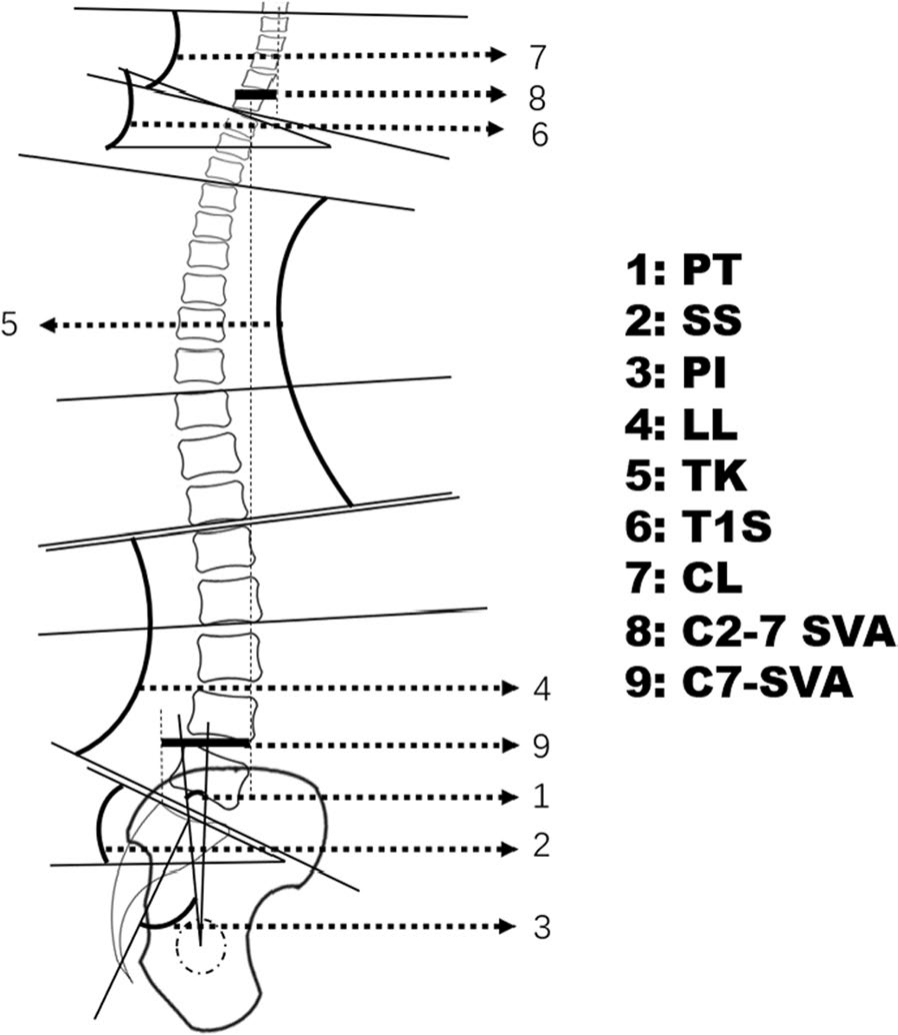
Method to measure spino-pelvic sagittal alignment. PT: pelvic tilt; SS: sacral slope; PI: pelvic incidence; TK: thoracic kyphosis; LL: lumbar lordosis; CL: cervical lordosis; T1S: T1 slope; SVA: sagittal vertical axis

Sagittal spino-pelvic parameters were measured using full-length X-rays taken in standing (Fig. 1). The C2–C7 Cobb angle (CL) is the angle between the lower endplate of the C2 vertebral body and the lower endplate of the C7 vertebral body. The C2–7 SVA is the axial distance from the center of the C2 vertebral body to the posterior upper of the C7 vertebral body. T1S is the angle between the horizontal plane and the T1 upper-end plate. Pelvic tilt (PT) is the angle between the line from the midpoint of the S1 upper endplate to the midpoint of the line connecting the center of the femoral head on both sides and the vertical line. Pelvic incidence (PI) is the angle between the line from the midpoint of the S1 upper endplate to the midpoint of the line connecting the centers of the femoral heads on both sides and the vertical line of the S1 upper endplate. Sacral slope (SS) is the angle between the S1 upper endplate and the horizontal line. Lumbar lordosis (LL) is the angle between the S1 upper endplate and the L1 upper endplate. Thoracic kyphosis (TK) is the angle between the T12 lower endplate and the T4 upper endplate. C7-SVA is the distance from the vertical line of the midpoint of the C7 vertebral body to the posterior superior of the sacrum. The change of cervical lordosis angle is the difference between the postoperative and preoperative CL. A change in the cervical lordosis angle ≥ 5° was defined as increased cervical lordosis. A change in the cervical lordosis angle ≤ –5° was defined as decreased cervical lordosis. A change in the cervical lordosis angle between –5° and 5° was defined as maintained cervical lordosis.

Images were viewed using our PACS software, UniWeb Viewer v7.0.1524 (EBM technologies, China). All parameters were measured and calculated by two spine surgeons who specialize in musculoskeletal disorders with more than 5 years of experience.

### Clinical parameters

Spinal cord neurological function was assessed according to the Japanese Orthopaedic Association (JOA) score, and the recovery rate was calculated according to the method described by Hirabayashi et al. [11]. The recovery rate (%) = (postoperative JOA − preoperative JOA)/(full score – preoperative JOA) * 100%. The recovery rate was used to evaluate the recovery of postoperative neurological function. The cervical function was scored using the neck disability index (NDI). The scores were recorded before surgery and during follow-up.

### Data analysis

All data were analyzed using IBM SPSS statistics 22.0 (IBM) or R software (v.4.2.2). The data were analyzed using the t test, Kruskal–Wallis H test, chi-square, receiver operating characteristic (ROC) curve analysis, one-way analysis of variance, and post hoc test. We performed decision curve analysis (DCA) by using the R package “dcurves.” The normal distributions were expressed as mean ± standard deviation. The non-normal distributions were represented by the median M (quartile). Differences with *P* ≤ 0.05 define statistical significance.

## Result

### Patient information

We included 56 patients, 29 males and 27 females, with an average of 62.39 ± 11.61 years. The follow-up time was at least 6 months (maximum 23 months) with an average of 13.4 ± 5.6 months. Ten patients underwent LP from the C3 segment, 43 from the C4 segment, and three from the C5 segment. The average CL angle after surgery was 14.13 ± 14.86°, which was –3.01 ± 12.99° compared to before surgery. The preoperative JOA score was 11.69 ± 2.51, and the postoperative JOA score was 13.95 ± 2.34. There were no decreases in neurological function scores. The preoperative NDI score was 15.56 (9.44, 31.00) %, and the postoperative NDI score was 15.00 (4.89, 24.89) %. The incidence of postoperative cervical kyphosis deformity was 19.64%. The recovery rate of JOA was 50.84 ± 29.03%.

No significant differences were found in the comparisons of spino-pelvic sagittal parameters before and after surgery. JOA score was significantly improved after surgery, and the NDI score showed no significant difference before and after surgery (Table 1).

**Table 1:** Spino-pelvic sagittal parameters and clinical data

Characteristics	Pre-surgery parameters	Post-surgery parameters	*P* value
PI (°)	46.29 ± 9.86	46.18 ± 9.67	0.761
SS (°)	25.50 (12.00, 34.45)	26.00 (11.73, 34.90)	0.138
PT (°)	20.70 (12.40, 29.38)	20.80 (12.55, 28.70)	0.346
LL (°)	34.26 ± 12.24	34.84 ± 11.64	0.246
TK (°)	35.67 ± 11.68	35.14 ± 10.86	0.238
T1S (°)	26.52 ± 6.11	25.79 ± 5.76	0.173
CL (°)	17.14 ± 10.18	14.13 ± 14.86	0.088
C7-SVA (cm)	3.65 ± 3.60	3.78 ± 3.53	0.551
C2–7 SVA (cm)	1.98 ± 1.17	2.14 ± 1.16	0.466
JOA score	11.69 ± 2.51	13.95 ± 2.34	** < 0.001**
NDI (%)	15.56 (9.44, 31.00)	15.00 (4.89, 24.89)	0.081

*PT* pelvic tilt, *SS* sacral slope, *PI* pelvic incidence, *TK* thoracic kyphosis, *LL* lumbar lordosis, *CL* cervical lordosis, *T1S* T1 slope, *SVA* sagittal vertical axis, *JOA* Japanese Orthopaedic Association, *NDI* neck disability index. Bold value indicates *p* value ≤ 0.05 and is statistically significant

### Analysis of related factors of cervical lordosis changes

The correlation analysis between the sagittal parameters of the spine and the changes in cervical lordosis revealed that the changes in cervical lordosis correlated with SS, PT, LL, T1S, and C7-SVA (*P* < 0.05) (Table 2). SS, PT, and LL weakly correlated with changes in cervical lordosis (Pearson correlation coefficients were 0.385, –0.338, and 0.348, respectively). T1S and C7-SVA moderately correlated with the changes in cervical lordosis after surgery (Pearson correlation coefficients were –0.419 and –0.591, respectively).

**Table 2:** Relationship between spino-pelvic sagittal parameters and cervical lordosis changes

		SS	PT	LL	TK	T1S	CL	C7-SVA	C2–7 SVA	Cervical lordosis changes
PI	Pearson coefficients	0.411**	0.339*	0.429**	− 0.173	− 0.127	− 0.062	0.018	− 0.105	0.078
	*P* value	**0.002**	**0.011**	**0.001**	0.202	0.353	0.652	0.897	0.442	0.569
SS	Pearson coefficients		− 0.718**	0.606**	− 0.166	− 0.182	− 0.090	− 0.415**	0.026	0.385**
	*P* value		**0.000**	**0.000**	0.222	0.179	0.510	**0.001**	0.848	**0.003**
PT	Pearson coefficients			− 0.298*	0.039	0.091	0.046	0.441**	− 0.107	− 0.338*
	*P* value			**0.026**	0.776	0.503	0.738	**0.001**	0.432	**0.011**
LL	Pearson coefficients				0.121	− 0.201	− 0.288*	− 0.479**	0.016	0.348**
	*P* value				0.373	0.138	**0.032**	**0.000**	0.905	**0.008**
TK	Pearson coefficients					0.312*	0.343**	− 0.137	− 0.117	0.035
	*P* value					**0.019**	**0.010**	0.315	0.392	0.799
T1S	Pearson coefficients						.508**	.481**	− 0.172	− 0.419**
	*P* value						**0.000**	**0.000**	0.205	**0.001**
CL	Pearson coefficients							0.442**	− 0.051	− 0.195
	*P* value							**0.001**	0.709	0.150
C7-SVA	Pearson coefficients								− 0.142	− 0.591**
	*P* value								0.297	**0.000**
C2–7 SVA	Pearson coefficients									0.046
	*P* value									0.734

^*^P < 0.05, **P < 0.01

*PT* pelvic tilt, *SS* sacral slope, *PI* pelvic incidence, *TK* thoracic kyphosis, *LL* lumbar lordosis, *CL* cervical lordosis, *T1S* T1 slope, *SVA* sagittal vertical axis. Bold value indicates *p* value ≤ 0.05 and is statistically significant

The patients are divided into a cervical lordosis reduction group, a cervical lordosis increase group, and a cervical lordosis unchanged group. There were 18 patients in the cervical lordosis reduction group, 15 in the cervical lordosis increase group, and 23 in the cervical lordosis unchanged group. There were significant differences in SS, PT, LL, T1S, and C7-SVA among the three groups before surgery (Table 3). Logistic regression analysis was performed to identify factors related to the reduction of cervical lordosis. T1S and C7-SVA were significantly related to the reduction of cervical lordosis (*P* = 0.021 and *P* = 0.001, respectively) (Table 4). There were significant differences in postoperative NDI score and JOA improvement rate among the three groups (*P* < 0.001 and *P* < 0.001, respectively). The outcomes in the cervical lordosis reduction group were poor.

**Table 3:** Comparison among groups of different cervical lordosis changes

	Cervical lordosis reduction (18)	Cervical lordosis increase (15)	Cervical lordosis unchanged (23)	*P* value
Age (years)	66.94 ± 10.16	58.80 ± 8.28	61.17 ± 13.02	0.106
Sex (male/female)	9/9	10/13	9/6	0.615
PI (°)	45.76 ± 13.16	46.01 ± 7.84	46.89 ± 8.35	0.931
SS (°)	11.50 (6.75, 27.73)	29.40 (14.00, 40.10)	26.80 (23.00, 37.30)	**0.010**
PT (°)	24.35 (22.40, 38.25)	17.00 (10.90, 26.60)	16.90 (11.80, 26.90)	**0.023**
LL (°)	27.89 ± 9.67	38.41 ± 12.40	36.53 ± 12.41	**0.022**
TK (°)	34.88 ± 10.04	35.34 ± 7.49	36.52 ± 15.02	0.901
T1S (°)	30.11 ± 4.16	22.53 ± 4.97	26.31 ± 6.53	**0.001**
CL (°)	19.28 ± 7.05	13.22 ± 11.90	18.02 ± 10.74	0.205
C7-SVA (cm)	6.85 ± 1.67	2.00 ± 3.83	2.22 ± 2.93	** < 0.001**
C2–7 SVA (cm)	2.10 ± 1.12	1.97 ± 0.92	1.99 ± 1.21	**0.937**
JOA recovery rate (%)	29.94 ± 19.11	57.56 ± 23.42	62.81 ± 30.64	** < 0.001**
Post-surgery NDI (%)	28.17 (17.50, 38.67)	11.56 (2.67, 20.44)	9.33 (0, 18.22)	** < 0.001**

*PT* pelvic tilt, *SS* sacral slope, *PI* pelvic incidence, *TK* thoracic kyphosis, *LL* lumbar lordosis, *CL* cervical lordosis, *T1S* T1 slope, *SVA* sagittal vertical axis, *JOA* Japanese Orthopaedic Association, *NDI* neck disability index. Bold value indicates *p* value ≤ 0.05 and is statistically significant

**Table 4:** Logistic regression for cervical lordosis reduction

		B	Standard error	Wald x^2^	*P* value	Exp (B)
Parameters	T1S	0.32	0.14	5.32	**0.021**	1.38
	C7-SVA	0.971	0.28	12.11	**0.001**	2.64

*T1S* T1 slope, *SVA* sagittal vertical axis. Bold value indicates *p* value ≤ 0.05 and is statistically significant

**Fig. 2:**
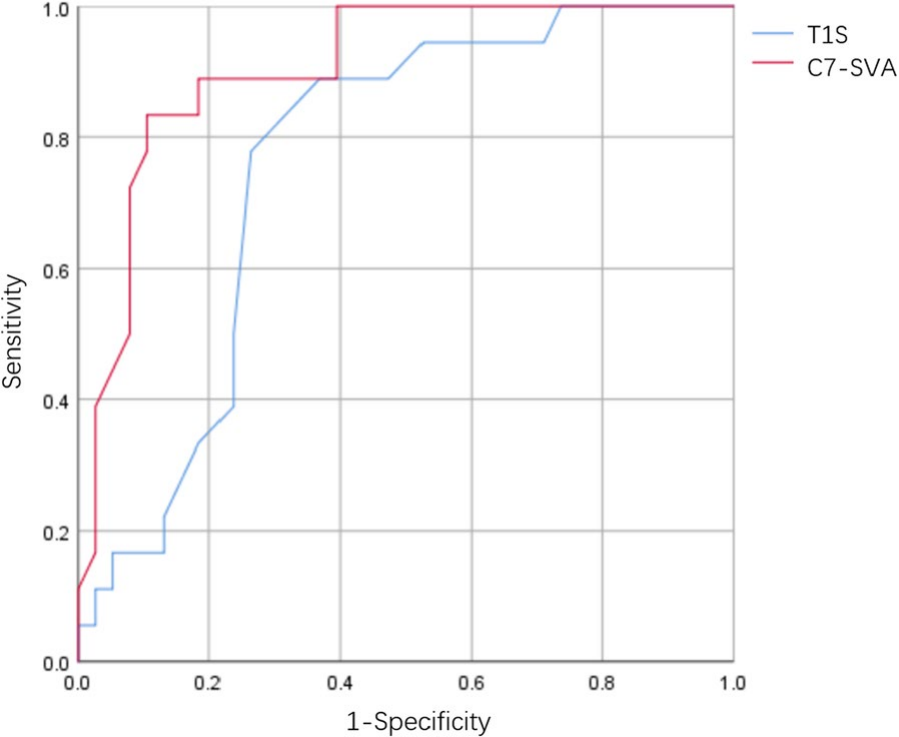
Receiver operating characteristic curve of T1S and C7-SVA predicting cervical lordosis loss. T1S: T1 slope; SVA: sagittal vertical axis

**Fig. 3:**
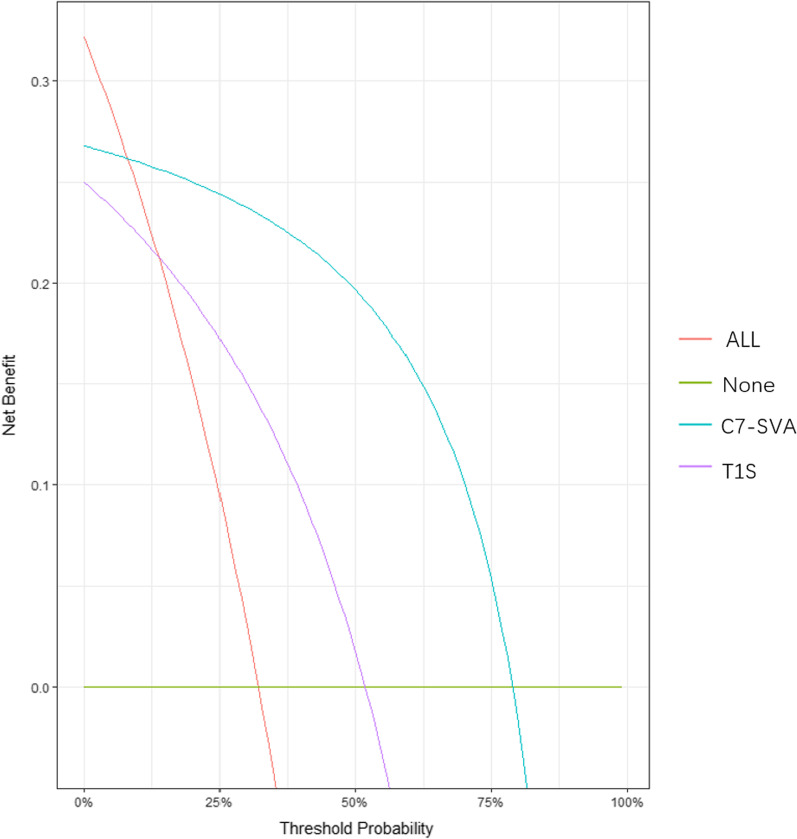
Decision curve analysis (DCA) diagram of C7-SVA and T1S predicting loss of cervical lordosis. T1S: T1 slope; SVA: sagittal vertical axis

ROC curve analysis was performed to determine the effectiveness of T1S and C7-SVA in predicting postoperative cervical lordosis loss (Fig. 2). T1S and C7-SVA predicted the loss of cervical lordosis (area under the ROC curve [AUC] of T1S = 0.760; the cutoff was 27.00, *P* = 0.002. The AUC of C7-SVA = 0.905; the cutoff value was 4.90, *P* < 0.001). Moreover, we performed decision curve analysis (DCA) and found that both C7-SVA and T1S could predict cervical lordosis loss, and C7-SVA was better than T1S to predict loss of cervical lordosis (Fig. 3).

### Comparison of different T1S and C7-SVA groups

According to the cutoff values of T1S and C7-SVA, patients were divided into four groups: a low T1S low SVA group, a high T1S low SVA group, a low T1S high SVA group, and a high T1S high SVA group. The comparison of sagittal spinal parameters before surgery in the four groups showed no significant difference in PI, TK, and CL among the four groups (*P* = 0.790, *P* = 0.226, and *P* = 0.083, respectively). There were significant differences in the changes of SS, PT, LL, and lordosis changes among the four groups (*P* < 0.001, *P* < 0.001, and *P* < 0.001, respectively). The loss of cervical lordosis was more significant in the high T1S high SVA group, and cervical lordosis was maintained better in the low T1S low SVA group. Cervical lordosis was lost less in the high T1S low SVA group and the low T1S high SVA group. For patients with more loss of cervical lordosis, the JOA recovery rate and postoperative NDI scores were poor (Table 5).

**Table 5:** Comparison among groups of different T1S and C7-SVA

	Low T1S, low SVA (21)	High T1S, low SVA (16)	Low T1S high SVA (8)	High T1S high SVA (11)	*P* value
Age (years)	59.95 ± 11.99	60.31 ± 10.45	65.50 ± 11.70	67.39 ± 11.60	0.222
Sex (male/female)	10/11	9/7	5/3	5/6	0.832
PI (°)	47.53 ± 8.02	46.45 ± 7.97	46.26 ± 12.94	43.72 ± 13.66	0.790
SS (°)	29.01 ± 13.64	30.73 ± 7.600	19.00 ± 10.16	7.73 ± 3.10	**< 0.001**
PT (°)	16.00 (10.50, 28.10)	15.90 (11.80, 20.15)	23.90 (20.90, 33.00)	29.80 (23.80, 55.00)	**< 0.001**
LL (°)	39.30 ± 11.07	40.35 ± 9.73	21.60 ± 10.58	24.98 ± 4.76	**< 0.001**
TK (°)	33.19 ± 9.68	40.73 ± 13.55	35.18 ± 11.07	33.43 ± 11.90	0.226
T1S (°)	20.77 ± 4.43	32.36 ± 2.83	25.38 ± 3.37	31.30 ± 4.12	**< 0.001**
CL (°)	12.88 ± 11.86	20.36 ± 11.00	21.18 ± 6.98	17.64 ± 5.63	0.083
C7-SVA (cm)	1.14 ± 3.29	2.52 ± 2.00	7.05 ± 1.20	7.61 ± 1.90	**< 0.001**
C2–7 SVA (cm)	2.24 ± 1.00	1.79 ± 1.12	1.78 ± 1.30	2.10 ± 1.02	0.718
Cervical lordosis change (°)	5.54 ± 4.70	− 1.12 ± 8.07	− 4.56 ± 16.33	− 20.96 ± 9.15	**< 0.001**
JOA recovery rate (%)	62.06 ± 24.88	55.46 ± 33.05	36.62 ± 28.91	33.02 ± 19.12	**0.018**
Post-surgery NDI (%)	7.01 ± 7.29	12.92 ± 9.12	21.26 ± 5.01	35.09 ± 10.00	**< 0.001**

*PT* pelvic tilt, *SS* sacral slope, *PI* pelvic incidence, *TK* thoracic kyphosis, *LL* lumbar lordosis, *CL* cervical lordosis, *T1S* T1 slope, *SVA* sagittal vertical axis, *JOA* Japanese Orthopaedic Association, *NDI* neck disability index. Bold value indicates *p* value ≤ 0.05 and is statistically significant

## Discussion

Studies found that when patients with cervical spondylosis undergo posterior cervical surgery, they are more likely to lose lordosis of the cervical spine because of damage to the posterior structures and paraspinal muscles [4, 12]. Larger T1S, larger C2–7 SVA, and smaller cervical lordosis are associated with cervical kyphosis deformity or loss of cervical lordosis after LP [3, 9, 13]. However, these studies omit an understanding of the correlation between global spinal sagittal parameters and the reduction of cervical lordosis after LP surgery. The results of the present study showed that there was a correlation between preoperative spino-pelvic sagittal parameters and postoperative cervical lordosis loss. Parameters reflecting lumbar degeneration (i.e., LL, PT, and SS) are weakly correlated with cervical lordosis loss, and parameters reflecting global spine sagittal balance (i.e., T1S and C7-SVA) are moderately correlated with the reduction of cervical lordosis after LP. T1S and C7-SVA are the critical factors for cervical lordosis change. We found that C7-SVA correlated with LL, PT, and SS, suggesting that patients with increased C7-SVA had lumbar degeneration, resulting in decreased LL and increased PT due to pelvic posterior rotation compensation. Thus, there was a weak correlation between lumbar degeneration and the change of cervical lordosis after cervical surgery. For patients with loss of cervical lordosis, the loss of lordosis and kyphosis deformity in the sagittal sequence of the cervical spine after surgery increases the mechanical stress in the front of the cervical spinal cord, which modulates the effect of surgical decompression and results in poor outcomes. Therefore, the JOA recovery rate and postoperative NDI score of patients with loss of cervical lordosis are poor.

Since Knott proposed T1S in 2010, its physical significance has been studied. T1S is related to the overall balance of the spine [14]. T1S increases in patients with a poor overall sagittal balance of the spine. The correlation between T1S and the reduction of lordosis after cervical surgery has been confirmed by many studies [9, 13]. Similarly, the present study found a correlation between large T1S and cervical lordosis loss and poor clinical efficacy after posterior laminoplasty. However, the present study included the overall sagittal parameters of the spine and considered that the increase of T1S may only be one reason for the loss of cervical lordosis, and the overall sagittal balance of the spine was also involved in the maintenance of cervical balance.

We found that patients with larger C7-SVA and larger T1S were more likely to have cervical lordosis loss. According to ROC curve analysis, we found that T1S and C7-SVA have good discriminant power, which predicts the loss of cervical lordosis (AUC = 0.760 and AUC = 0.905, respectively). DCA was used to compare the efficacy of C7-SVA and T1S predictive models to maximize the clinical benefits when false positives and false negatives are known to be unavoidable [15–17]. DCA also confirmed that T1S and C7-SVA could predict the loss of cervical lordosis and showed that C7-SVA was a better predictor than T1S.

**Fig. 4:**
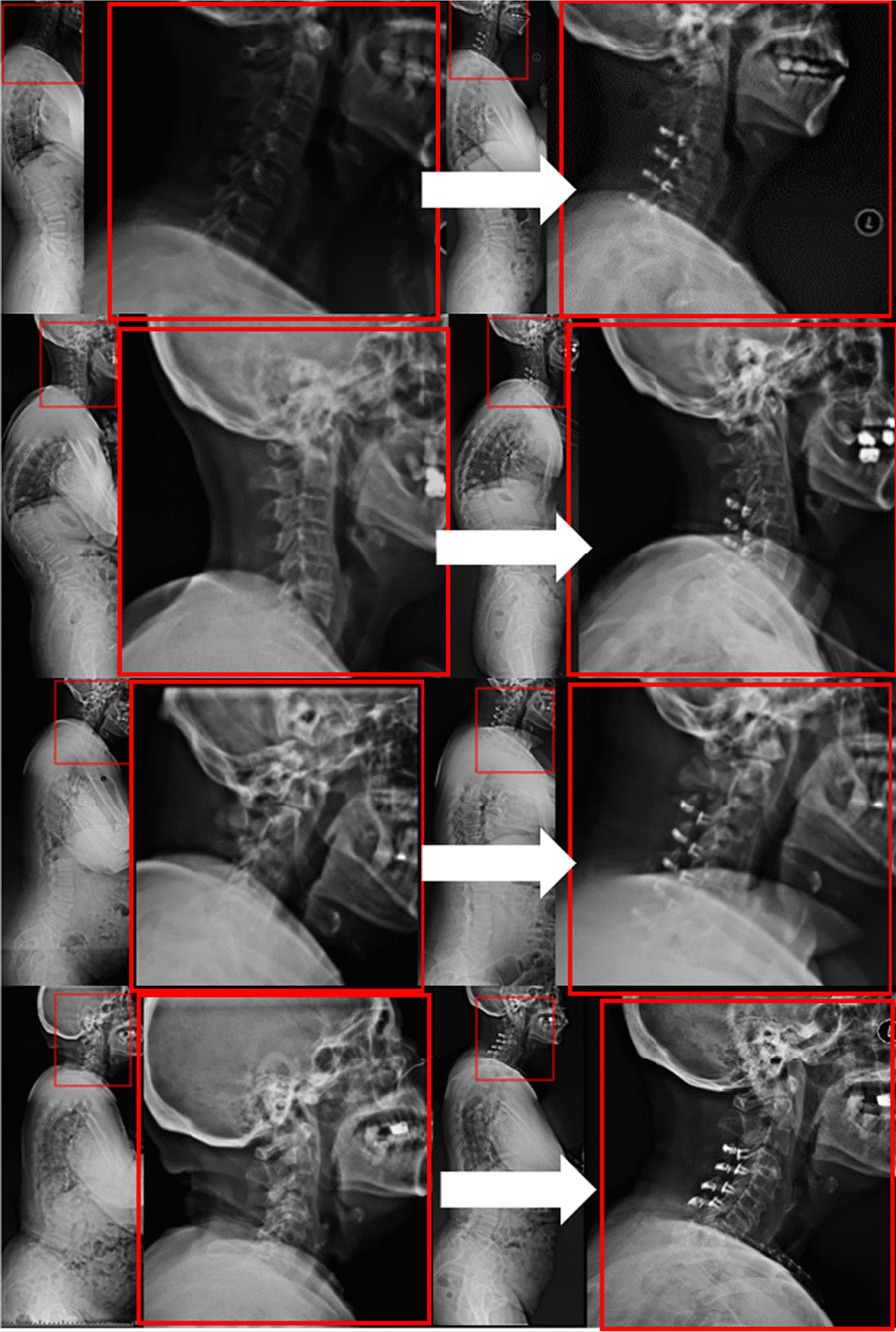
Full-length spinal films and enlarged cervical spine images in the low T1S low SVA group, the high T1S low SVA group, the low T1S high SVA group, and the high T1S high SVA group. Compared with the other three groups, the patients with high T1S and high C7 SVA had significant loss of cervical lordosis after surgery. T1S: T1 slope; SVA: sagittal vertical axis

We then evaluated the effects of different T1S and C7-SVA groups on cervical sagittal alignment after LP. For patients with low T1S and low SVA, the cervical spine and spine are in overall balance. The destruction of paracervical muscles after LP in this group causes less damage to the balance of the cervical spine. However, for patients with high T1S and high SVA, the cervical extensor dorsalis is essential in maintaining cervical vertebra posterior extension and cervical lordosis. The damage to the posterior structure breaks the balance of anterior and posterior forces of the cervical vertebra; therefore, this group of patients is often more prone to postoperative loss of cervical lordosis. The sagittal balance of the spine in the low T1S and high SVA groups was affected. After the overall imbalance, although thoracic compensation failed to change the overall balance of the spine, the compensation did not lead to an excessive increase in T1S, and the local balance of the cervical spine was partially maintained. The cervical lordosis was lost less in this group. The overall sagittal balance of the spine in the high T1S and low SVA groups was better. The increase in T1S might be related to fundamental anatomical factors or local kyphosis; however, the spine is compensated, and a small amount of cervical lordosis is lost less after surgery (Fig. 4).

This study shows that the LL of patients with increased C7-SVA was smaller than that of patients with normal C7-SVA. The decrease in LL was related to the changes in lumbar degeneration. In elderly patients, the lumbar spine may be degenerated (including kyphosis and disk degeneration), resulting in whole-spine imbalance [18]. For sagittal spine imbalance patients, pelvic extension and thoracic kyphosis reduction compensatory mechanisms occurred to maintain the sagittal balance of the spine [19]. In the present study, the increase in C7-SVA, the increase in PT, and the decrease in SS indicate that the pelvic supination compensation occurs to compensate for the damage to the overall sagittal balance of the spine. However, there was no significant difference in TK among the four groups. This finding might be related to the difficulty of thoracic compensation in the degeneration of paravertebral muscles in elderly patients. However, the sample size was small, which might impact the results. Nevertheless, we found that pelvic compensation was more common in elderly patients with sagittal spinal imbalance.

In addition to cervical spine degeneration, the degeneration of other parts of the spine affects the overall sagittal balance and affects the sagittal balance of the cervical spine. For patients whose overall sagittal balance of the spine is damaged (because the sagittal balance of the cervical spine is also often damaged), internal fixation and fusion surgery may be considered to maintain the postoperative cervical balance after posterior cervical surgery [20–22]. These findings suggest that, for patients with cervical spondylosis, preoperative evaluation of the overall sagittal parameters of the spine can determine whether the patient is in a state of spinal degeneration and spine balance change. The evaluation of the overall sagittal balance of the spine is critical for the prediction of postoperative cervical lordosis loss and the selection of surgical methods.

There are some limitations to this study. The number of patients was relatively small, and the follow-up period was short; larger sample sizes and longer follow-up intervals are required to validate our findings. There is a lack of lower limb compensation data supporting the compensation mechanism. We only considered the sagittal parameters and did not consider factors such as whether the cervical muscles were atrophic and weak. Nevertheless, this study provided theoretical support for the relationship between the full-length sagittal parameters of spine and changes in cervical sagittal alignment after LP.

## Conclusion

Patients undergoing cervical LP surgery require evaluating the sagittal balance of the cervical spine and the whole spine. T1S and C7-SVA correlated with changes in cervical sagittal alignment after LP surgery. C7-SVA was better at predicting the loss of cervical lordosis than T1S.

## Data Availability

All data generated or analyzed during this study are included in this published article and its supplementary information files.
